# Expression of Concern: Dexmedetomidine postconditioning suppresses myocardial ischemia/reperfusion injury by activating the SIRT1/mTOR axis

**DOI:** 10.1042/BSR-2019-4030_EOC

**Published:** 2022-07-27

**Keywords:** Dexmedetomidine, EX527, mTOR, Myocardial ischemia/reperfusion, SIRT1

The Editorial Office has been made aware of potential issues surrounding the scientific validity of this paper, hence has issued an Expression of Concern to notify readers whilst the Editorial Office investigates. Duplications have been identified between this paper and another by Feng et al. 2017 (“Protective effect of tanshinone IIA against cardiac hypertrophy in spontaneously hypertensive rats through inhibiting the Cys-C/Wnt signaling pathway” DOI: 10.18632/oncotarget.14328). Two sections of the MI/R+Dex+EX527 panel of Figure 6b appear to be the same as sections in figure panel 2c of the Feng et al. paper, one of which is horizontally flipped. The authors have been contacted with regards to these concerns.

